# Determinants of COVID-19 vaccination decision among Filipino adults

**DOI:** 10.1186/s12889-023-15712-w

**Published:** 2023-05-10

**Authors:** Lourdes Marie Sequerra Tejero, Rosemary Ruiz Seva, Bettina Joyce Petelo Ilagan, Kattleea Lorezca Almajose

**Affiliations:** 1 Technology Transfer and Business Development office, University of the Philippines Manila, Manila, Philippines; 2 Industrial and Systems Engineering, De La Salle University, Manila, Philippines; 3 College of Arts and Sciences, Cavite State University, Indang, Cavite, Philippines; 4 Lyceum of the Philippines University Manila, Manila, Philippines

**Keywords:** COVID-19, Vaccination, Logistic regression, Vaccine acceptance

## Abstract

**Background:**

With a number of vaccines against COVID-19 now widely available globally, it is opportune to determine what tips the decision to get vaccinated. In most countries like the Philippines where the government provides these vaccines for free to all its citizens, their COVID-19 vaccine awareness and COVID-19 information sources as well as their socio-demographic profile were considered as primary factors that could possibly affect vaccination decisions. Participants’ income level was considered as a possible financial consideration that can affect vaccination decision as transport to vaccination sites might entail costs to them.

**Methods:**

This study used a cross sectional survey design wherein participants came from all regions of the Philippines. An online questionnaire was voluntarily answered by Filipinos aged 18–80 years of age.

**Results:**

A total of 2,268 participated in the survey with 1,462 having complete responses which were included in the analysis. Those who are younger, with higher educational attainment, with public health insurance, with employers requiring vaccination, high awareness about COVID-19 vaccination, and high vaccine confidence are more likely to get vaccinated. On the other hand, those with long-standing illness and those residing outside the national capital region are less likely to get vaccinated.

**Conclusion:**

Vaccination decisions among Filipinos are determined by their age, educational attainment, health insurance, employer requirement, high awareness of the disease, and a high level of vaccine confidence.

**Supplementary Information:**

The online version contains supplementary material available at 10.1186/s12889-023-15712-w.

## Introduction

On March 11, 2020, the World Health Organization (WHO), in a media briefing opening remarks by its director-general, characterized COVID-19 as a pandemic. While the development of vaccines is deemed as one of the most important responses to curb this global health crisis, WHO emphasized that it is vaccination per se, and not the vaccines that will put an end to this pandemic. However, WHO also acknowledged that there are challenges to ensure that people around the world get vaccinated.

In January 2021, the Department of Health of the Philippines issued an interim plan for the deployment of COVID-19 vaccines [1]. The strategy was that of a whole-of-society approach with the government leading the deployment of vaccines and implementation of the vaccination program. Of the 110 million population, about 70 million were considered eligible for the vaccines for 2021, based on pre-identified groupings.

Sallam [2] found out that vaccine hesitancy, or the “delay in acceptance or refusal of vaccination despite availability of vaccination services”[3], is considered as a common phenomenon globally. He furthers that, in his review of COVID-19 vaccine acceptance rates worldwide, vaccine hesitancy can be the major hindrance of the control efforts to lessen the negative consequences of COVID-19 pandemic, at least in certain countries/regions.

In the Philippines, decisions to get vaccinated, particularly of the COVID-19 vaccine, are driven by Filipinos’ concerns about having more information about the various vaccines available to them [4]. United Nations Development Programme (UNDP) Philippines, in August 2021 reported that the facilitating factors for vaccination included concern for family and loved ones, COVID-19 risk perception, approval of an endorsement by either the Food and Drug Administration (FDA) or the Department of Health (DOH), and work-related reasons; while barriers to vaccination included concerns about side-effects, medical reasons, news about vaccines, and vaccine effectiveness and efficacy [5]. It was mentioned in this same report that COVID-19 vaccination in the Philippines started in March 2021 and that by August 15, 2021, around 40.4 to 44.1% of the adult population have received at least one COVID-19 vaccine shot. The Philippine government’s initial target was 70% of the population by the end of 2021.

Despite these findings, however, several factors affecting decisions to get vaccinated against COVID-19, given the multifaceted nature of vaccine hesitancy [2] and the history of vaccine uptake in the Philippines marked by critical challenges [6] required further studies.

Hence, this quantitative, correlational research on the determinants of COVID-19 vaccination decision among Filipino adults. As transport to vaccination sites might entail costs for people, participants’ income level was considered as a financial factor that can affect their vaccination decision. Non-financial factors considered were socio-demographic characteristics; COVID-19 information sources, awareness, and worry; and vaccine confidence and functional health literacy. The study examined if both these financial and non-financial factors could determine participants’ vaccination decision.

## Methods

### Research design

This is a cross sectional survey design wherein participants from all the 17 administrative regions of the Philippines were included in the sample. Following the formula provided by Bujang, et al. [7], the required number of participants is 850.

Data collection was conducted in the first quarter of 2022, the time when a recent surge of cases was declining early in that quarter [8], and when the Philippine government is nearing its target of vaccinating 70% of its population against COVID-19 [9]. It was opportune at this time to investigate the drivers for actual vaccination. Participants of the study were Filipinos aged 18–80 years old, currently residing in the country, were qualified to take the COVID-19 vaccine, could read, and understand Filipino and/or English, and could answer the questionnaire online.

Two questionnaires were developed in two languages: English and Filipino. The English questionnaire was translated from English to Filipino by a qualified translator. The English questionnaire was piloted first to 25 individuals to ensure the clarity of questions. Corrections were made on the format of the questionnaire and order of questions. After translation, the Filipino questionnaire was piloted to another 25 people. The Filipino version was revised to simplify the difficult Filipino terms used in the first version.

The survey was administered online using two methods: first is self-administered (91%) and the second was by a trained research assistant interviewer (9%) for those who were in remote areas and had difficulty accessing the Internet. The main survey questionnaire administration was conducted between March to April of 2022. Participation in the study was voluntary. Recruitment was done through social media platforms and through email. Participants were given an online survey link and they had the option to answer either a questionnaire in English or a questionnaire in Filipino. Answering the questionnaire and subsequently submitting it, constituted participant’s consent in the study. Those who requested reimbursement for the internet services incurred in answering the online questionnaire were reimbursed accordingly. The responses to the questionnaire remained anonymous since the request for reimbursement came in separately from the questionnaire. All the questions in the questionnaire were mandatory.

### Measures

A questionnaire was designed ad hoc to collect the data for this research. The sociodemographic data included age, sex, educational attainment, employment status, income level, health insurance, health status, and region of residence.

Employer requirement for vaccination and advice by a health care provider to vaccinate against the disease are binary variables in the model where a yes answer was coded as 1. Information sources included three items that identify a person’s social context - family and friends, co-workers, and healthcare workers. Nine items that describe their information environment - government agencies, TV, newspaper, radio, Facebook, Instagram, Twitter, TikTok, and YouTube. The frequency of access followed a five-point Likert scale measured as (1) never, (2) rarely, (3) sometimes, (4) often, and (5) always. For COVID-19 vaccination awareness, participants were asked to answer a 9-item quiz-type survey with statements formulated based on information presented on the websites of the Philippines’ Department of Health and the United States of America’s Center for Disease Control and Prevention. The three levels of awareness were given scores as follows: 1 (correct information), 0 (unaware of the information), and − 1 (wrong information) in the model.

COVID-19-related worry was determined using a three-item scale taken from Head et al., [10] to measure participants’ personal worry about COVID-19. A 5-point Likert-type scale was used where 1 = strongly disagree to 5 = strongly agree.

Participants’ vaccine-related profiles included information on *functional health literacy* (FHL) and vaccine confidence. All four items to measure FHL were taken from Biasio et al., [11]. As for *vaccine confidence*, five items were adapted from the 8-item Vaccine Confidence Scale by Gilkey et al., [12] measured using a 10-point sliding scale from 0-strongly disagree to 10-strongly agree because of their relevance to study objectives. The 5 items included were: (1) Vaccines are necessary to protect health, (2) Vaccines do a good job in preventing the diseases they are intended to prevent, (3) Vaccines are safe, (4) If I get vaccinated there can be serious side effects and, (5) In general, medical professionals in charge of vaccinations have my best interest at heart. Item 4 was reverse coded.

Participants’ vaccination status is a binary variable where one refers to getting the full dose of the vaccine or at least one shot of a two-dose vaccine. Their vaccination status equated to their vaccination decision, considered as the dependent variable in the model.

### Data analysis

Aside from age, all sociodemographic data were considered categorical variables in the model. Coded data was processed using MS Excel and scored. Responses with scores were summed and used in calculating the total scores of vaccination awareness, COVID-19-related worry, FHL, and vaccine confidence. Sociodemographic data were summarized using a frequency table. Quantitative data were presented as mean and standard deviation. Binary logistic regression (BLR) was performed to determine predictors of COVID-19 vaccine decision. The BLR model was constructed by including all 15 covariates at the same time. A full model was constructed that included all the variables in the analysis. This approach was used to prevent bias in the selection of variables to be included in the model.

Data were analyzed using SPSS 21.0 (IBM Corp.: Armonk, NY, USA). Significance level considered was 0.05.

### Ethics statement

All methods were carried out in accordance with relevant guidelines and regulations. Since all participants were 18 years old and above, the consent of a guardian was not required. The introductory letter to participants in the survey questionnaire contained relevant information about the research including objectives, benefits to the participant, anonymity, expected time for their participation, utilization of data gathered, contact information of the researchers, etc. It also indicated that participation was voluntary and continuing to answer the survey was an expression of consent. The study protocol, survey questionnaire, including ‘waiver of informed consent documentation’ were reviewed and approved by the University of the Philippines Manila’s Review Ethics Board, with code UPMREB 2021-0673-01.

## Results

A total of 2,268 participated in the survey from which 1,462 complete survey responses were recorded and analyzed. Incomplete responses were not included in the data analysis. A response is considered incomplete if the respondent failed to finish the survey.

### Participants’ sociodemographic characteristics

Table 1 summarizes the sociodemographic characteristics of the 1,462 participants in the study. When data gathering was conducted, around 70% of the Philippine population had already been vaccinated. Participants came from all the regions in the Philippines but the distribution of the sample did not follow the actual geographical distribution. 61% (61%) of the participants came from densely populated areas in the Philippines, such as CALABARZON (25%), National Capital Region (20%), and Central Visayas (16%). Participants aged 21–30 comprised the highest percentage of the sample (36%), followed by those younger than 21 (20%). People older than 60 comprised 6% (6%) of the sample only. 69% (69%) of the sample were female. Approximately two-thirds (66%) were college graduates, and 54% were employed. More than half (52%) belong to the poor and low-income group, and 47% have no health insurance. Eighty one percent (81%) do not have a long-standing illness.

**Table 1:** Sociodemographic Characteristics of Participants

Sociodemographic Characteristics	Frequency	Percentage
Age		
<=20 21–30 31–40 41–50 51–60 61–70 71–80	291 528 215 196 154 55 23	20 36 15 13 11 4 2
Sex		
Male Female	449 1,013	31 69
**Educational Attainment**		
Elementary to high school College Postgraduate	156 959 347	11 65 24
**Employment Status**		
Unemployed and retired Government/private/self-employed Student	531 785 146	36 54 10
**Income Level**		
Poor Low income Lower middle income Middle income Upper middle income High income and rich	514 243 329 187 114 75	35 17 23 13 8 5
**Health Insurance**		
None Public Private Both private and public	692 368 254 148	47 25 17 10
**Health Status**		
Without long-standing illness With long-standing illness	1186 276	81 19
**Region of Residence**		
National Capital Region Luzon Visayas Mindanao	290 619 307 246	20 42 21 17

### Participants’ COVID-19-related Profile

Most (86%) of the participants were either partially or fully vaccinated. Seventy one percent (71%) received a recommendation from a healthcare professional to get a COVID-19 vaccine. Of the 785 employed participants, 83% were required by their employers to get vaccinated against the disease.

As can be seen in Table 2, the average awareness score is 2.81 indicating that participants know at least 3 correct COVID-19 vaccine information. Details of the COVID-19 Awareness data were included in Annex 2. Majority (93%) are aware that the vaccines are free, that they come in different brands (89%) and they are effective in helping protect against severe disease and death (83%). However, around 96% are not aware that vaccines have side effects that are normal and believe that it contains microchips that can alter DNA (89%).

The mean FHL score indicates a general difficulty understanding published information about COVID-19 vaccines. The participants are highly worried about getting infected by the virus but have high levels of confidence in the vaccine.

**Table 2:** Participants’ COVID-19-related Profile

COVID-19 Related Profile (Scale)	Mean	Standard Deviation
**COVID-19 Vaccination Awareness Score**	2.81	1.97
**COVID-19-Related Worry Score**	11.85	2.72
**Functional Health Literacy Score**	10.35	2.93
**Vaccine Confidence Score**	36.53	10.33

Table 3 summarizes the sources and frequency of access to COVID-19 vaccination information. The top three most often accessed sources of information on COVID-19 vaccination are Facebook, TV, and family and friends.

**Table 3:** COVID-19 Vaccination Information Sources

INFORMATION SOURCES	MEAN	RANK	FREQUENCY OF ACCESS
Facebook	3.90	1	Often
TV	3.70	2	Often
Family and friends	3.50	3	Often
Government agencies	3.40	4	Sometimes
Healthcare workers	3.20	5	Sometimes
YouTube	2.70	7	Sometimes
Co-workers	2.70	7	Sometimes
Radio	2.70	7	Sometimes
Newspaper	2.40	9	Sometimes
Instagram	2.20	10.5	Rarely
Tiktok	2.20	10.5	Rarely
Twitter	2.10	12	Rarely

### Sociodemographics, COVID-19-related Profile, and vaccine-related Profile Associated with Vaccination decision

The results of the binomial logistic model of the sociodemographic, COVID-19-related profile and information sources are presented in Table 4. Logistic regression analysis was performed to explore the influencing factors associated with the decision to get vaccinated against the COVID-19 virus. Younger age (aOR = 0.94, 95% CI = 0.92–0.96), higher educational attainment (aOR = 5.25, 95% CI = 2.75–10.03; aOR = 6.22, 95% CI = 2.14–18.13), those with public health insurance (aOR = 2.47, 95% CI = 1.23–4.96), those with employers requiring vaccination (aOR = 4.28, 95% CI = 2.18–8.43), high awareness about COVID-19 vaccination (aOR = 1.22, 95% CI = 1.11–1.34), and high vaccine confidence (aOR = 1.15, 95% CI = 1.12–1.19) are more likely to get vaccinated. Those with long-standing illness tend not to get vaccinated (aOR = 0.38, 95% CI = 0.19–0.76). As for the region of residence, those regions outside the national capital region (NCR) are less likely to get vaccinated.

**Table 4:** Sociodemographics, COVID-19-related profile and information sources associated with vaccination decision according to binomial logistic regression

Characteristics	Adjusted OR	95% CI	p value
**Age**	0.944	0.924–0.964	*< 0.001*
**Sex**			
Male	ref.	ref.	ref.
Female	1.379	0.797–2.387	0.250
**Education**			
Less than college	ref.	ref.	ref.*
College	5.252	2.750-10.031	*< 0.001*
Postgraduate	6.220	2.135–18.125	0.001
**Employment Status**			
Unemployed	ref.	ref.	ref.
Employed	1.157	0.585–2.287	0.675
Student	1.083	0.481–2.435	0.847
**Family Income**			
Poor	ref.	ref.	ref.
Low income	0.565	0.275–1.161	0.120
Lower middle income	0.756	0.330–1.727	0.506
Middle income	1.652	0.428–6.381	0.467
Upper Middle income	1.300	0.320–5.284	0.714
High income	3.610	0.329–39.661	0.294
**Health Insurance**			
None	ref.	ref.	ref.
Public	2.466	1.226–4.960	0.011
Private	0.909	0.414–1.995	0.811
Public and private	0.985	0.324–2.991	0.979
**w/ long-standing illness**	0.379	0.189–0.761	0.006
**Region**			
NCR	ref.	ref.	ref.
Luzon	0.371	0.150–0.917	0.032
Visayas	0.172	0.061–0.480	0.001
Mindanao	0.147	0.054–0.401	*< 0.001*
**Vaccination as employer requirement**	4.283	2.176–8.433	*< 0.001*
**Healthcare provider advising vaccination**	1.234	0.717–2.125	0.447
**Awareness on COVID-19 vaccines**	1.217	1.108–1.336	*< 0.001*
**COVID-19 Vaccination Information Sources**	1.004	0.979–1.029	0.781
**COVID-19 worry**	1.051	0.958–1.152	0.291
**Functional Health literacy**	1.019	0.937–1.108	0.665
**Vaccine Confidence**	1.152	1.120–1.185	*< 0.001*

## Discussion

Several socio-demographic variables significantly affect the vaccination decision of participants in the sample. Results suggest that young participants are more likely to take the vaccine as the proportion of unvaccinated individuals increases with age. This is consistent with previous findings that young people are less hesitant to receive the COVID19 vaccine [13, 14]. During the survey, health authorities in the Philippines struggled to vaccinate its elderly population because they do not turn up at the vaccination sites, meeting only 5% of the target during a three-day vaccination drive [15]. One of the reasons cited by health authorities is the belief of older adults that they do not have many years to live, making the vaccine unnecessary[16]. Some elderly people are also difficult to reach, especially those living in rural areas [16].

Higher education levels such as college and postgraduate degrees, largely determine vaccination decisions in this study. A closer look at the data showed vaccine awareness and functional literacy levels are lower for those without college degrees. Literature on vaccine determinants worldwide consistently showed that fewer years of education decreases the chance of COVID19 vaccine uptake [17, 18]. People with higher education have heightened awareness of the risks and benefits of the vaccine because they have more knowledge about the vaccine and the vaccination process. Similarly, high vaccine acceptance rates among college students in China were associated with high knowledge [19].

Sex, employment status, and family income were unrelated to the study’s vaccination decision, probably because the Philippine government made COVID-19 vaccines free for all its citizens; thus, financial incapacity and unemployment did not deter anyone from getting vaccinated. Men and women received the vaccines to comply with institutional requirements. Our study showed that employers requiring COVID-19 vaccination increased Filipinos’ likelihood of vaccinating. The Presidential announcement requiring vaccination for employees working onsite [20] strongly influenced the people’s decision to get the shots. For the unvaccinated, their options were to work from home or undergo regular RT-PCR testing as prescribed by the Philippine Department of Labor and employment [21]. With the high costs of tests, employees generally opted to get vaccinated.

Geographic location has a strong association with vaccination uptake since the Philippines is an archipelago consisting of more than 7,000 islands. Delivering the vaccines to far-flung areas from the National Capital Region (NCR) is a logistical challenge that contributes to the difference in vaccination rates across areas. During the time of the vaccine rollout, local government units (LGUs) interviewed from Luzon, Visayas, and Mindanao expressed concerns about vaccine handling, especially the need for cold storage facilities and vehicles to transport the vaccines [22]. Aside from logistical problems, the Geographically Isolated and Disadvantaged Areas (GIDA) in the Philippines are also affected by the pandemic response due to communist terrorist groups in the area [23].

The participants came from all the regions of the country although the distribution of the sample did not reflect the actual geographical distribution. The participants lived mainly in highly urbanized areas whereas the Philippines has more rural communities. NCR is the center of the country’s economic activity and the largest of all metropolitan areas. The likelihood of getting vaccinated decreases as the region gets further away from the NCR as urbanization significantly determines the vaccine acceptance rate [24]. In Canada, more people were vaccinated in large metropolitan areas, just like the NCR [24]. Moreover, population density is generally higher in urban areas, especially in the NCR, making the people more concerned about disease transmission.

Vaccine awareness of participants in this study is generally high as most lived in urban areas. There was high awareness in NCR and Luzon. People who live far from the capital and/or urban areas may have less access to information materials and modalities, hence the lower vaccination uptake in these areas. Two common mistakes emerged from our results about COVID19 vaccines. Most participants believed that they do not have to take the full dose of the vaccine and that vaccines can alter or change their DNA, turning them into genetically modified human beings. The false information about DNA modification lowered vaccine acceptance and was found to be one of the most common themes in a study of 52 countries about vaccine rumors and conspiracy theories [25]. Taking only the first of a 2-dose COVID-19 vaccine gives protection for a limited period. The second dose prolongs the duration of this protection by activating the helper T cells of the immune system [26].

Participants who know about vaccine brands, compositions, and doses, as well as vaccination effectiveness and side effects after vaccination being considered normal, have a higher tendency to decide to get vaccinated than those with little or no knowledge of these things. Nomura et al. [27] also found that people’s perceptions of the risks and benefits of a COVID-19 vaccine were significantly associated with their vaccination intention. These findings imply that for vaccination intention and eventually vaccination decision to increase, people should be made aware of the benefits of availing the COVID-19 vaccine.

Having misconceptions about the vaccine strongly correlated with the study participants’ not getting vaccinated. This is corollary of their most frequently cited source of information which was Facebook where fake news abound [28]. Moreover, the moderate level of functional health literacy of the participants reflects the difficulties they may have in discerning false from true information. Thus, more effective and targeted information dissemination schemes are needed to address this situation.

People with long standing illness were not likely to get vaccinated against COVID 19. Vaccine hesitancy remains to be an issue among those with health problems. Reluctance is due to lack of understanding, fear of adverse effects, and negative COVID-19 Vaccination information [16]. In the Philippines, more than a quarter of those aged 35–59 years have been diagnosed with illnesses that predispose them to serious effects of COVID-19 [29]. With the community quarantine focusing more on these high-risk groups, they are more susceptible to such fear and reluctance.

The level of vaccine confidence among the participants is high. Those who indicated that the COVID-19 vaccines are safe and needed to protect one’s health were more likely to get vaccinated. The lowest confidence rating was related to the vaccine’s side effects, followed by doubt about its safety. Findings of similar studies in Japan and South Korea showed that the most cited reasons for low vaccine confidence were side effects and that vaccines had not been sufficiently tested [30]. The decline in the COVID19 vaccine confidence in the Philippines may have been influenced by the Dengvaxia vaccine fiasco in 2017. The controversy caused the vaccine confidence of Filipinos to decline from 93% to 2015 to 32% in 2018 [31]. Traumatic experiences concerning previous vaccination and information obtained from traditional, social media, and neighbors further contribute to delay and refusal of vaccination [32].

Those who have public health insurance are more likely to get vaccinated. Since the COVID-19 vaccination program for the whole Philippines is government-funded and controlled, those with public health insurance may be more familiar with the public health system and may be able to access the COVID-19 vaccination program.

The results of this study have implications for the public health approach to increasing COVID-19 vaccinations. Since the younger ones are more inclined to get vaccinated, they may serve as the conduit to reach the older ones, especially the hesitant people with chronic illnesses, as they are more at risk of contracting COVID-19. The government can increase the vaccination rate of older people by offering packages to incentivize young people taking their older parents for vaccination. Since the lines for vaccination are usually long, a young person together with a person aged more than 50 years old may be in the priority lane.

Results also indicate that the educated and those with higher awareness levels of COVID-19 are more likely to get vaccinated. This is consistent with the findings of previous studies [33, 34]. Since the community quarantine is easing up and people can go out of their homes, information, education, and communication (IEC) materials should be designed for the less educated people and be situated in locations where these people frequent like the streets, bus stops, and the like. Around 72% of Filipinos have access to a smartphone [35] so the government can possibly tie up with telecommunications companies to spread crucial information through text messages about COVID-19 vaccination and correct false information from social media. This can be especially effective for those in the rural areas where information through the media may be harder to reach. Thus, for rural areas the IEC materials may be placed in locations and settings where people usually congregate, like in the market, plaza, municipal hall, and the like.

Study findings showed that participants actively sought for COVID-19 vaccination information from Facebook, TV, and family and friends almost every week. This result implies that there is a need for concerned officials to fill their Facebook pages and posts with information related to their respective COVID-19 vaccination programs. In this case, individuals who can acquire these pieces of information can share these with family and friends who seek the same from them.

The study should also provide an impetus for the government to provide the infrastructure and equipment for better vaccine deployment for COVID-19 and other potential disease outbreaks in the future. The US Agency for International Development pledged USD 315 million for cold chain facilities and mobile vaccination sites to reach far flung areas in the Philippines [36]. This is a short-term solution to address current needs; however, the government has to strategically plan and identify supply chain solutions at the national level such as providing roads, bridges, and basic infrastructure especially to underdeveloped areas.

This study’s results are comparable to the findings in countries near the Philippines. In Malaysia where the government also purchased their vaccines against COVID, about two-thirds of the respondents were willing to get vaccinated. They were those from the lower age group, those with higher education, females, and not having chronic disease. Moreover, the strongest drivers for their decision to get vaccinated were vaccine effectiveness and suggestions from their Ministry of Health [37]. In China on the other hand, those who were older, had a lower education level, lower income, higher trust in the vaccine and higher perceived risk of infection showed a higher probability to vaccinate [38]. There is a greater proportion of older people in China than in the Philippines or Malaysia which may explain the differing results in terms of age. There are also differences in literacy rate which may account for the different results. What is common among these countries is that the perceived effectiveness of the vaccine is the main driver for people to get vaccinated.

The present study was conducted in the second year after the start of the pandemic when the Philippines had gone through peaks of the COVID-19 infection that claimed thousands of lives. The vaccine seemed to be the only hope to avoid deaths. Moreover, people needed to work for their sustenance. Even those who were previously hesitant to get vaccinated, did so because it was required by their employers [20]. Thus, the proportion of those who got vaccinated is high. As the threat of COVID-19 waned with the increased herd immunity, there is a decreased uptake of the booster doses of the vaccine [39]. It is foreseen that there may be an eventual decline in the uptake of the biannual booster shots. Unless there is an effective campaign not only from the medical community but more importantly from the government, people will not take the succeeding doses of the vaccine. This study offers some directions in devising campaign strategies based on the factors influencing decision to vaccinate against COVID-19.

Limitations for this present study include using purposive sampling in recruiting participants instead of probability sampling because of the constraints brought about by the pandemic situation. The link to the survey was sent to contacts and institutions all over the country through electronic means. Researchers ensured that respondents came from all the 17 regions in the country, representing the different categories of the main variables of this study. There were difficulties in finding unvaccinated individuals as the proportion of the vaccinated went over 80% in the NCR. With the collaboration of enumerators from different regions, an adequate number of unvaccinated people were eventually included in the study, approximating the proportions in the regional levels. Potential bias in the collection of data by enumerators from various regions may have happened but this was minimized with the proper orientation of the enumerators.

Online questionnaires naturally are accessible by those with the appropriate gadgets and internet access, thus potentially limiting the inclusion of those with low resources. To mitigate this, the researchers offered to refund the internet fees incurred by respondents. Moreover, the enumerators aided those who were old, illiterate, and without gadgets to record their responses on their own devices. Thus, there were some respondents from these categories who were able to participate in this study.

## Conclusion

Based on the findings of the study, it can be concluded that among Filipino adults, COVID-19 vaccination decision is determined by their age, educational attainment, health insurance, and employer requirement. Further, it can be concluded that a high awareness of the disease and a high level of vaccine confidence correlates with the decision to get vaccinated.

Accessing the true information about the disease and vaccine is key in reaching the decision to get vaccinated. It also contributes to high levels of vaccine confidence. Thus, effective information dissemination schemes targeted according to the socio-demographic profile, health literacy and sources of information of the intended audience will result in better vaccine uptake.

## Electronic supplementary material

Below is the link to the electronic supplementary material.

Supplementary Material 1

## Data Availability

The datasets used and/or analyzed during the current study are available from the corresponding author on reasonable request.
