# Supplementary material for: Determinants of COVID-19 vaccination decision among Filipino adults
Source: BMC Public Health. 2023 May 10;23:851. doi: 10.1186/s12889-023-15712-w (PMC10170431; doi:10.1186/s12889-023-15712-w)
Supplement: Supplementary file 1 — Supplementary Material 1 [file 12889_2023_15712_MOESM1_ESM.docx]

Annex 1. Questionnaire

**A. Sociodemographic Characteristics**

Birthdate: Month _______ Year________

Sex: ______ Male ______ Female ______ Prefer not to say

Highest educational attainment (check your highest educational attainment, whether graduated or not in that level)

___Elementary

___High School

___College

___Masters

___PhD

Please indicate your Profession:

___ healthcare professional/worker (pls indicate) ________________

___ non-health related (pls indicate)___________________

Employment status:

___unemployed

___employed (if employed, check all items below that apply to you)

___employed in government agency

___employed in private company

___self-employed

___others: (please indicate) _____________________

Does your employer require you to get vaccinated against COVID-19?

___ Yes ____ No ___ not applicable (i.e., not employed)

Health status:

___ with diagnosed chronic illness

___ without any chronic illness

Location of residence: ___________________(indicate city or municipality and province)

Health insurance:

___none

___private

___public

___private and public

___others (please specify)_________________________________

Monthly Family Income: ________ Below P10,957

________ P10,957 to P21,914

________ P21,914 to P43,828

________ P43,828 to P76,66

________ P76,669 to P131,484

________ P131,483 to P219,140

________ Above P219,140

**B. COVID-19 Vaccination Information Sources: How often do you access these sources for information about COVID-19 vaccination?** (Check the boxes that applies for each item.)

Never Rarely Sometimes Often Always

(1 – 2 times (3 – 4 times (5-6 times (Everyday)

a week) a week) a week)

Significant other ________ ________ ________ ________ ________

Family and relatives ________ ________ ________ ________ ________

Friends ________ ________ ________ ________ ________

Co-workers ________ ________ ________ ________ ________

Healthcare workers ________ ________ ________ ________ ________

Government channels_______ ________ ________ ________ ________

National TV ________ ________ ________ ________ ________

Local TV ________ ________ ________ ________ ________

National Newspaper ________ ________ ________ ________ ________

Local Newspaper ________ ________ ________ ________ ________

Radio ________ ________ ________ ________ ________

Facebook ________ ________ ________ ________ ________

Instagram ________ ________ ________ ________ ________

Twitter ________ ________ ________ ________ ________

TikTok ________ ________ ________ ________ ________

YouTube ________ ________ ________ ________ ________

Others: please specify:

_______________ ________ ________ ________ ________ ________

**C. COVID-19 Vaccination Awareness: What information about COVID-19 vaccination have you acquired from the sources identified in Part B?** (Tick the box that corresponds to your answer.)

| **Statements** | **Not aware** | **Disagree** | **Agree** |
| --- | --- | --- | --- |
| COVID-19 vaccines differ in their compositions and come in different brands. |  |  |  |
| COVID-19 vaccines are effective at helping protect against severe disease and death. |  |  |  |
| There may be side effects after COVID-19 vaccination and these are not normal. |  |  |  |
| Everyone aged 12 years and older are recommended to get vaccinated. |  |  |  |
| COVID-19 vaccines are available in the Philippines for everyone at no cost. |  |  |  |
| It typically takes two weeks after vaccination for the body to build protection (immunity) against the virus that causes COVID-19. |  |  |  |
| People may not receive all recommended doses of a COVID-19 vaccine. |  |  |  |
| COVID-19 vaccines contain microchips and can change or alter my DNA. |  |  |  |
| There are priority groups in the Philippine government’s COVID-19 vaccination program. |  |  |  |
| There is an ongoing COVID-19 vaccination program in our community that I can avail if I want to. |  |  |  |

**D.** **Functional Vaccine Literacy**

When reading or listening to information about COVID-19 vaccines,

| **Statements** | **Often** | **Sometimes** | **Rarely** | **Never** |
| --- | --- | --- | --- | --- |
| Did you find words you didn’t know? |  |  |  |  |
| Did you find that the texts or images were difficult to understand? |  |  |  |  |
| Did you need much time to understand them? |  |  |  |  |
| Did you or would you need someone to help you understand them? |  |  |  |  |

**E. COVID-19 related worry**

| **Statements** | **Strongly**  **Disagree** | **Disagree** | **Neither agree**  **Nor disagree** | **Agree** | **Strongly**  **Disagree** |
| --- | --- | --- | --- | --- | --- |
| I am scared about getting infected with COVID-19 |  |  |  |  |  |
| The possibility of getting infected in the future with COVID-19 concerns me |  |  |  |  |  |
| I don’t really worry about getting infected with COVID-19 |  |  |  |  |  |

**F. Health provider recommendation for vaccine:**

*“Has a doctor or other healthcare professional ever recommended that you receive COVID-19 vaccine?”*

___Yes

___No

___Others (please specify) _________________________________________

#

**G. Vaccine Confidence**

The following items describe statements about your level of confidence on the COVID-19 vaccine. Please indicate your agreement or disagreement by ticking the appropriate circle using the scale.

|  | Disagree Strongly Agree |
| --- | --- |
| Vaccines are necessary to protect health. | O O O O O O O O O O O |
| Vaccines do a good job in preventing the diseases they are intended to prevent. | O O O O O O O O O O O |
| Vaccines are safe. | O O O O O O O O O O O |
| If I get vaccinated there can be serious side effects. | O O O O O O O O O O O |
| In general, medical professionals in charge of vaccinations have my best interest at heart. | O O O O O O O O O O O |

Annex 2

Detailed Results on Awareness Questions

| No | COVID-19 Awareness Questions | % Correct |
| --- | --- | --- |
| 1 | COVID-19 vaccines differ in their compositions  and come in different brands. | 89 |
| 2 | COVID-19 vaccines are effective at helping  protect against severe disease and death. | 83 |
| 3 | There may be side effects after COVID-19  vaccination and these are not normal. | 96* |
| 4 | Everyone aged 12 years and older are recommended  to get vaccinated. | 74 |
| 5 | COVID-19 vaccines are available in the  Philippines for everyone at no cost. | 93 |
| 6 | It typically takes two weeks after vaccination  for the body to build protection (immunity) against the virus that causes  COVID-19. | 75 |
| 7 | People may not receive all recommended doses of  a COVID-19 vaccine. | 57* |
| 8 | COVID-19 vaccines contain microchips and can  change or alter my DNA. | 89* |
| 9 | There are priority groups in the Philippine  government’s COVID-19 vaccination program. | 75 |

*reverse coded
